# Resistivity-Temperature Behavior of Intrinsically Conducting Bis(3-methoxysalicylideniminato)nickel Polymer

**DOI:** 10.3390/polym12122925

**Published:** 2020-12-06

**Authors:** Evgenii Beletskii, Valentin Ershov, Stepan Danilov, Daniil Lukyanov, Elena Alekseeva, Oleg Levin

**Affiliations:** Institute of Chemistry, St. Petersburg State University, 199034 Saint Petersburg, Russia; belochkin@yandex.ru (E.B.); valentin.ershov2015@yandex.ru (V.E.); stepandroid007@gmail.com (S.D.); lda93@yandex.ru (D.L.); alekseeva_ev@yahoo.com (E.A.)

**Keywords:** salen polymer, thermostability, conductivity, positive temperature coefficient

## Abstract

Materials with a positive temperature coefficient have many applications, including overcharge and over-temperature protection in lithium-ion (Li-ion) batteries. The thermoresistive properties of an electrically conductive polymer, based on a Ni(salen)-type backbone, known as polyNiMeOSalen, were evaluated by means of in situ resistivity measurements. It was found that the polymer was conductive at temperatures below 220 °C; however, the polymer increased in resistivity by three orders of magnitude upon reaching 250 °C. Thermogravimetric results combined with elemental analyses revealed that the switch from the insulation stage to the conductive stage resulted from thermally dedoping the polymer. Electrochemical studies demonstrated that a polymer retains its electroactivity when it is heated and can be recovered to a conductive state through oxidation via electrochemical doping in an electrolyte solution.

## 1. Introduction

The development of electronic and transportation technologies has increased the demand for energy storage at secondary chemical power sources. According to experts, the global amount of stored energy has increased significantly since 2005, and it is expected that the current amount will be doubled by 2025 [1]. Lithium-ion (Li-ion) batteries are among the most important energy storage technologies for mobile devices and electric vehicles due to their high energy density and reasonable power density [1,2]. Lithium-ion batteries contain extremely strong oxidizing and reducing agents as well as flammable organic electrolytes (the electric and magnetic fields (EMF) of such batteries may exceed 4 volts (V), and the charging voltage of traditional batteries are always more than 4.2 V). Therefore, safety concerns appear to be the most significant problem in the development of a new generation of lithium-ion batteries. Currently, approximately 20 incidences of fires or explosions as the result of lithium-ion batteries are registered every year [3]. As a result, the number of accidents and their residual consequences have become the most significant risks for both consumers and manufacturers.

One option for increasing the safety of Li-ion batteries is to modify battery electrodes with elements that have a positive temperature coefficient (PTC). For this purpose, several composites made of crystalline polymers with conductive fillers, e.g., polyethylene with carbon black [4,5], polyethylene with nickel [6] and epoxy resin with carbon [7], have been developed. The mechanism of action of such composites is based on the rupture of conductivity tracks inside the polymer matrix caused by the volumetric expansion of the crystalline polymer upon heating. The fault tolerance of these elements depends on their accuracy in maintaining a compound’s composition and homogeneity. As an alternative, PTC elements with conductive polymers, such as poly(3-butylthiophene) [8], poly(3-dodecylthiophene) [9] and poly(3-octylthiophene) [10], have been considered. These elements are switched into a conductive state by partial oxidation due to the formation of mobile, delocalized, positively charged quasiparticles, or polarons, compensated by counterion doping. The PTC effect of such elements is caused by the well-known thermal dedoping phenomenon. Thermal dedoping is an immanent property of a conductive polymer; thus, PTC elements with conductive polymers show a higher fault tolerance compared to the composites of crystalline polymers with conductive fillers. In this regard, the development of PTC electrically conductive polymers, which are stable in the operating conditions of Li-ion batteries, is an important task in the creation of safer Li-ion batteries.

**Figure 1:**
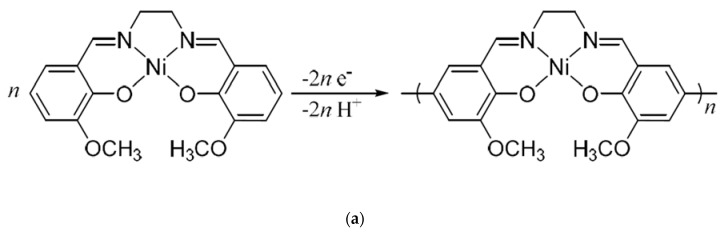

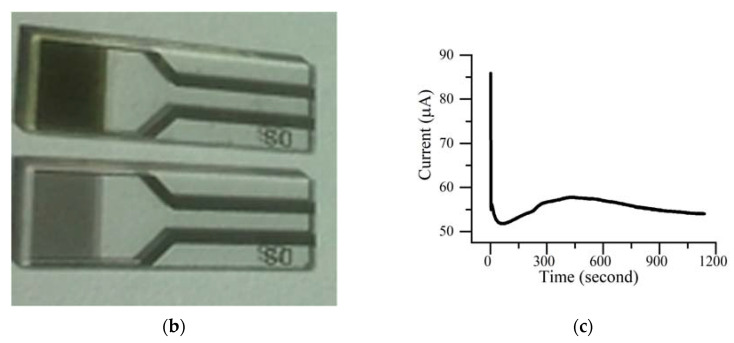
The scheme of the electropolymerization reaction: (**a**) polymer coated (left) and pristine (right) IDE electrodes and (**b**) polymer electrodeposition current versus time at a potential of 850 mV in a 5 mM monomer solution in acetonitrile with 0.1 M LiBF_4_ (**c**).

Here, we report the study of the PTC behavior of a polymeric complex made of nickel polyNiMeOSalen (Figure 1a). This polymer belongs to a family of Ni(salen)-type polymers, which are considered to be the electrode components in Li-ion batteries [11,12,13,14]. X-Ray photoelectron spectroscopy (XPS), scanning electron microscopy (SEM) coupled with an energy-dispersive X-Ray spectroscopy (EDX), synchronous thermal analysis (STA) and in situ conductivity measurements in an electrolyte solution were used to trace chemical and physical changes in the polymer upon heating.

## 2. Materials and Methods

### 2.1. Materials

The polyNiMeOSalen film, doped by BF_4_^−^ anion, was prepared by electrochemical polymerization from a 5 mM solution of monomers in 0.1 M LiBF_4_ (Sigma-Aldrich, St. Louis, US) in acetonitrile (HPLC grade, Kriochrom, St. Petersburg, Russia) in a glove box filled with argon (Villitek, Moscow, Russia, water content was less than 1 ppm). The polymerization scheme is presented in Figure 1a. Electropolymerization was carried out in a potentiostatic mode at a potential of 850 mV on the surface of an interdigitated platinum electrode (IDE) (G-IDEPT5, Metrohm, Herisau, Switzerland) using Elins P-40X potentiostat (Elins, Moscow, Russia) until 80 mC of charge was passed through the cell. This corresponded to a polyNiMeOSalen layer thickness of 2.65 μm, which was calculated from the electron numbers for the polymerization reaction and the density of polyNiMeOSalen, as reported in [15]. The geometry of the IDEs is shown in Figure 1b. The length of the electrode lines was 6.76 mm, the width of the lines was 5 μm, the gap between the lines was 5 μm, the number of lines was 250 and the electroactive area was 0.34 cm^2^. The Pt plate (1 cm^2^) was used as an auxiliary electrode. Reference electrode MF-2062 (Bioanalytical systems, West Lafayette, IN, USA) was used and consisted of a silver wire immersed in a 5 mM solution of AgNO_3_ in acetonitrile with 0.1 M (Et_4_N)BF_4_ (Sigma-Aldrich, St. Louis, MI, USA). The synthesis current versus the synthesis time is shown in Figure 1c. After synthesis, the films were washed with acetonitrile and dried in a glove box for one day.

### 2.2. Temperature Tests

An IDE electrode with attached leads was placed in a deep Teflon flask, into which a K-type thermocouple and a Teflon tube as an argon inlet were inserted. The thermocouple was located near the electrode surface. The flask was closed with a lid that had a small opening for argon outward flow. The argon flow rate was 0.2 dm^3^/min and the heating rate was 3 °C/min.

**Figure 2:**
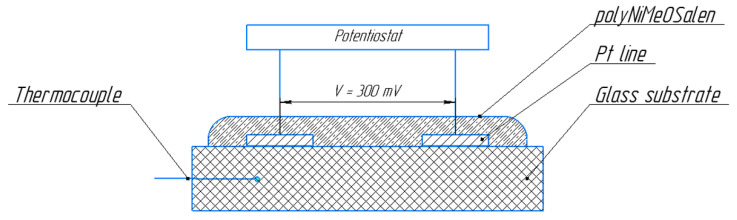
The wiring diagram of the potentiostat and thermocouple for temperature testing.

The temperature dependence of the polyNiMeOSalen resistance was measured in a potentiostatic mode in accordance with the scheme in Figure 2. A voltage of 300 mV was set between the combs’ termini, and the shunting current was recorded using an Elins P-40X potentiostat (Elins, Russia). Potential switching and the onset of temperature recording were synchronized in time. To record the temperature, a UNI-T UT325 digital thermometer (Uni-T, China) was used.

### 2.3. In Situ Conductivity Measurements in Solution

To measure conductivity, a polymer film was deposited on the IDE electrode as described above. The electrode was washed with acetonitrile, and its conductivity was determined in the electrolyte, which consisted of acetonitrile with 0.1 M LiBF_4_, before and after temperature testing. Film thickness was calculated based on its weight, which was derived from the charge consumed during the deposition ( 80 mC), the number of electrons of the monomeric unit [15], the electrode surface area of 0.34 cm^2^ and the polymer density determined previously [15]. IDE electrodes were used and consisted of the following geometry: a single track length of 6.76 mm, a single track width of 5 µm, an array band gap of 5 µm and 250 tracks for each working electrode. Cyclic voltammetry at 5 mV s^−1^ was performed on the IDE grids alongside two working electrodes with a constant difference of 10 mV between them. The currents passing through the working electrodes included the Faraday currents of the electrochemical process and the leakage Ohmic current. We assumed that the magnitude of the Faraday currents was the same on both working electrodes and that the leakage currents had the same magnitude $I_{l}$ and opposite signs for each working electrode. We could then determine the leakage current, and, according to Ohm’s law, the polymer resistance (or conductance). A more detailed description of this procedure is given in [16].

During potential scanning, the current flowing through the working electrodes WE-1 and WE-2 involved the Faradaic current of the electrochemical process and the leakage current:

(1) $$\begin{array}{c} I_{WE-1}=I_{F}+I_{l}\\ I_{WE-2}=I_{F}-I_{l} \end{array}$$

The measured difference between currents that were registered on the first and the second working electrodes $\Delta I=I_{WE-1}-I_{WE-2}$ were used to calculate the leakage current according to Equation (1) as

(2) $$I_{l}=\frac{I_{WE-1}-I_{WE-2}}{2}=\frac{\Delta I}{2}$$

Given the leakage current and the electrode parameters, we calculated the conductance (G) or resistance (*R*) of the polymer layer:

(3) $$G=\frac{1}{R}=\frac{\Delta I}{2V}$$

where V is the potential difference between the working electrodes.

Conductivity and resistivity were calculated according to [17]:

(4) $$\sigma =\frac{1}{\rho }=\frac{\Delta I}{2V}\cdot \frac{d}{hlf}$$

where σ is conductivity, ρ is resistivity, d is the distance between the working electrodes, h is the thickness of the film, f is the number of IDE lines and l is their length.

### 2.4. Characterization

Fourier-transform infrared spectroscopy (FTIR) with an attenuated total reflection (ATR) module was recorded on a Thermo Nicolet 8700 (Thermo Fisher Scientific, Waltham, MA, USA) spectrometer.

X-ray photoelectron spectroscopy (XPS) was carried out using Thermo Fisher Escalab 250Xi (Thermo Fisher Scientific, Waltham, MA, USA) with non-monochromatic AlKα radiation (photon energy 1486.6 eV). The total energy resolution of the experiment was about 0.3 eV. The spectra of the samples were recorded in a constant pass energy mode at 20 eV, using a 650 micron diameter analysis area. During data processing of the XPS spectra, binding energy values were referenced to the C1s peak (284.8 eV) from the adventitious contamination layer. Investigations were carried out at room temperature in an ultrahigh vacuum at the order of 10^−9^ mbar.

SEM images were obtained on a Zeiss Merlin (Carl Zeiss microscopy GmbH, Jena, Germany) microscope.

Simultaneous thermal analysis (STA) was performed in argon flow (0.2 dm^3^/min) on a NETZSCH STA 449 F3 STA449F (Netzsch GmbH & Co. KG, Selb, Germany) calorimeter with a heat rate of 5 °C/min and a temperature range of 20 to 800 °C.

## 3. Results and Discussion

### 3.1. Temperature Dependence

**Figure 3:**
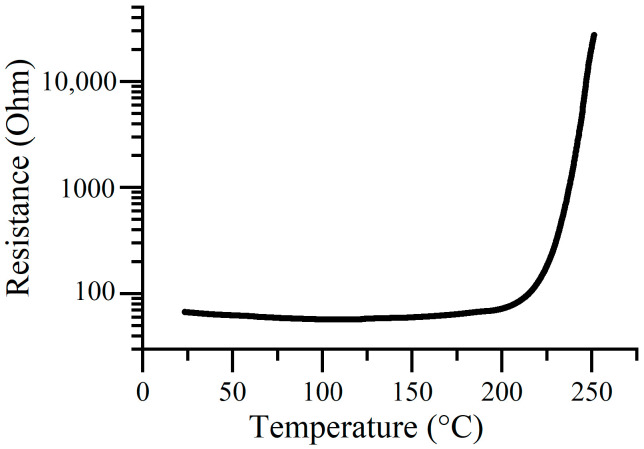
The temperature dependence of the electrical resistance of IDE with doped polyNiMeOSalen.

Figure 3 shows the temperature dependence of the electrical resistance of an IDE electrode with polyNiMeOSalen doped by BF_4_^−^. The doped polymer showed a fairly low electrical resistance at temperatures up to 220 °C, which indicated that the polymer is thermally stable in a temperature range of 20 to 220 °C. Moreover, at temperatures of 20 up to 125 °C, a gradual decrease in resistance was observed, which is typical of semiconductors [18,19,20]. Further heating was accompanied by slow resistance growth until 220 °C, at which point, there was a sharp increase by about three orders of magnitude.

**Figure 4:**
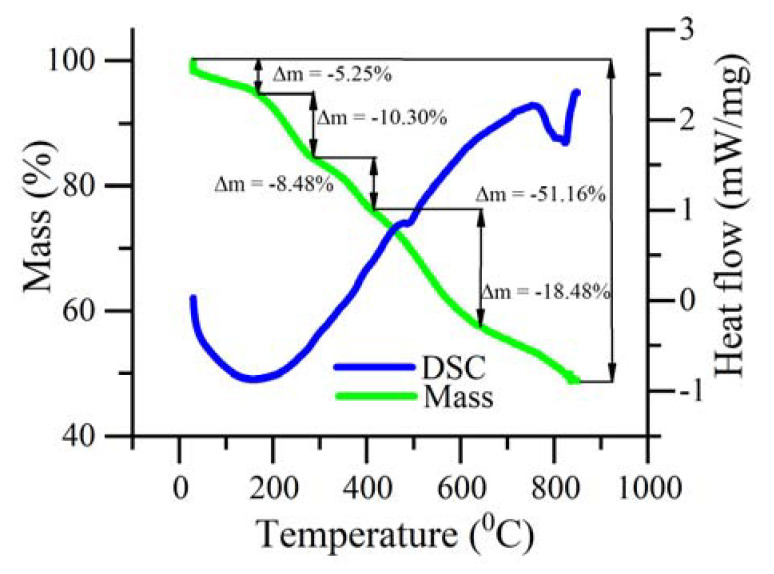
TGA (green line, left axis) and DSC (blue line, right axis) curves of doped polyNiMeOSalen in the temperature range of 20 to 850 °C.

Figure 4 shows the thermal gravimetric analysis (TGA) and differential scanning calorimetry (DSC) curves of BF_4_^−^ doped polyNiMeOSalen. Weight loss began within the temperature range where the polymer had a low resistance value. The first inflection point was situated at 150 °C with a weight loss of about 5%, which corresponded to the evaporation of the residual solvent. Up to a temperature of 220 °C, a loss of about 10% occurred. This loss was attributed to the dedoping process, i.e., the loss of the dopant (BF_4_^−^). Further heating to 850 °C led to an overall weight change of 51% and was associated with carbonization. ATR-FTIR spectra of the polymers before and after heating showed only a minor difference, which indicated the preservation of the chemical structure of the monomeric units (Supplementary Materials, Figure S1).

**Figure 5:**
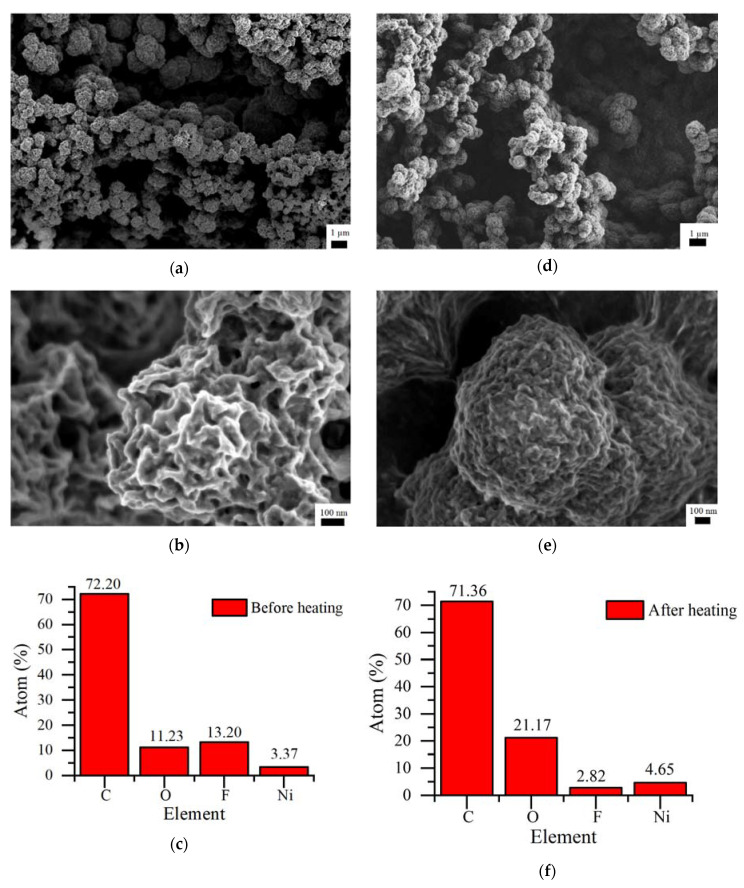
SEM/EDX results of the same samples, recorded before heating (**a**–**c**) and after heating to the temperature of 250 °C (**d**–**f**).

The morphology of the polymer films before and after thermal treatment is shown in Figure 5a–c. SEM images with low (Figure 5a,b) and high (Figure 5c,d) magnification showed little change in the polyNiMeOSalen film morphology after heating. In both cases, the samples had a globular structure with a globule size of about 0.5 μm. However, before heating, the globules’ edges were sharp, and the pores inside them were quite distinguishable. After heating, the globules looked denser and more rounded. The elemental composition of the film before and after heating it to 250 °C was estimated using EDX analysis (Figure 5e,f). The polymer backbone may be distinguished by nickel and oxygen signals. Carbon is a component of both the polymer and the solvent, which may have been trapped in the polymer film. The presence of fluorine indicated polymer doping with the BF_4_^−^ anion. The stochiometric C:O:Ni atomic ratio in the undoped polyNiMeOSalen was 18:4:1. An experimental C:O:F:Ni atomic ratio in dry polymer film prior to heating was found to be 21.4:3.3:3.9:1, which is close to the stoichiometry for nickel and oxygen. However, the ratio showed an excess of carbon and fluorine atoms, which can be explained by the presence of BF_4_^−^ together with the solvent molecules (acetonitrile). After heating, the C:O:F:Ni ratio changed to 15.4:4.6:0.6:1, which almost coincided with the stoichiometry of the undoped polymer. This indicated nearly completed thermal dedoping upon heating, with the loss of anions together with solvent molecules.

### 3.2. XPS Analysis

**Figure 6:**
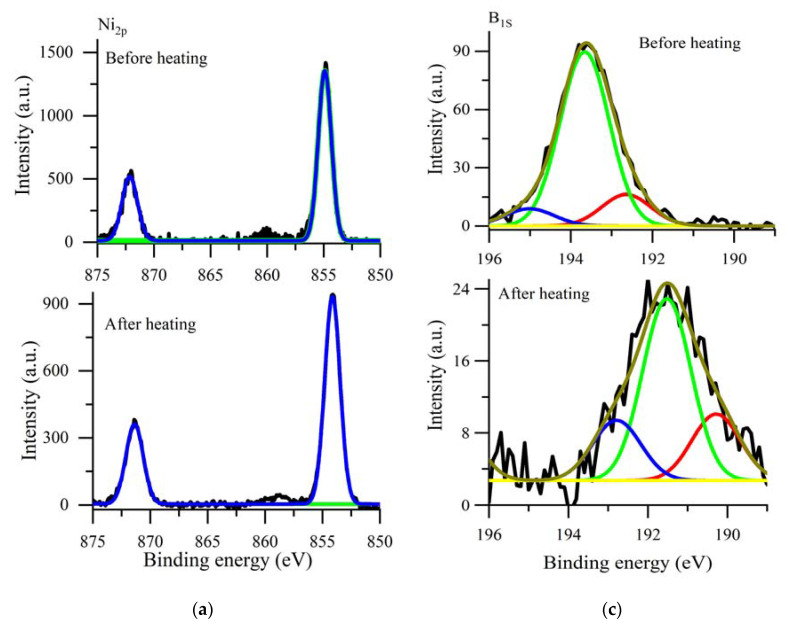

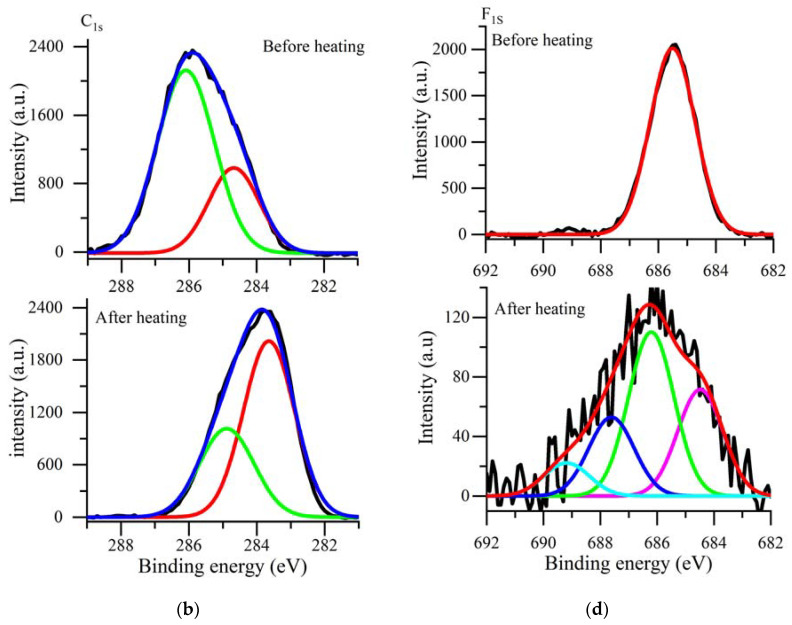
XPS spectra of poly[Ni(CH_3_OSalen)] at the binding energies, corresponding to Ni_2p_ (**a**), C_1s_ (**b**), B_1s_ (**c**), F_1s_ (**d**). (Black lines are the experimental results; colored lines are the results fitted with Gaussian).

The Ni_2p_ spectrum of the freshly prepared polyNiMeOSalen (Figure 6a) contained two peaks at 872.1 and 854.98 eV, typical for Ni(salen) complexes, which were assigned to Ni_2p1/2_ and Ni_2p3/2_ [21]. The C_1s_ spectrum (Figure 6b) of the same polymer contained two peaks at 286.1 and 284.1 eV, which are also typical for the C_1s_ spectrum of Ni(salen)s [21]. Intensities of the Ni and C lines in the spectrum of the sample after heating did not change significantly. The binding energies of all peaks on the Ni_2p_ spectrum as well as at the C_1s_ spectrum shifted less than 1 eV after heating.

The B_1s_ and the F_1s_ spectra (Figure 6c and d) contained the main peaks at 193.8 eV and 685.5 eV, respectively, which may have been assigned to BF_4_^−^ ions intercalated into the polyNiMeOSalen film during electrochemical doping. The spectra showed significant changes after heating. The intensities of the B_1s_ lines had decreased by four times and the intensity of the F_1s_ line had decreased by more than 10 times.

According to the XPS data, we can conclude that heating does not lead to the destruction of the polyNiMeOSalen backbone. However, during the heating, most of BF_4_^−^ ions were removed from the film. Upon analysis of the peak shape and the position on the B_1s_ and the F_1s_ spectra, obtained after heating, we can assume that the residual traces of B and F resulted from the BF_4_^−^ transformation to BF_3_^−^ as a result of heating [22].

### 3.3. Cyclic Voltammetry and Conductivity

**Figure 7:**
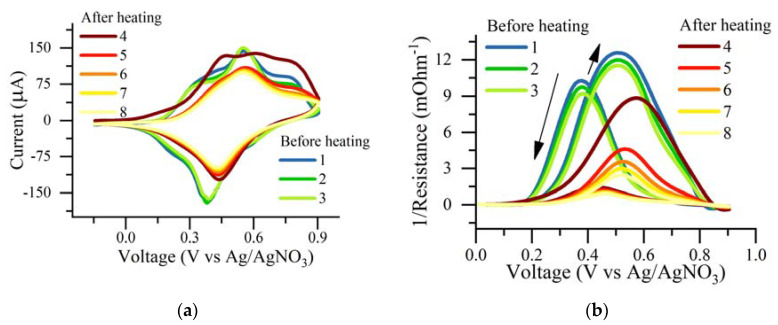
CVA (**a**) and in situ conductivity data (**b**) of polyNiMeOSalen before and after heat treatment. The electrolyte is 0.1 M LiBF4 in CH3CN and the scan rate is 5 mV/s.

The reversibility of the doping process of polyNiMeOSalen was probed by cyclic voltammetry and in situ conductivity measurements before and after heating. PolyNiMeOSalen was oxidized in 0.1 M LiBF_4_ at potentials higher than 0.2 V versus Ag/AgNO_3_. Polymer conductivity growth occurred until the oxidation peak potential was reached, the polymer was half-doped and the charged polymer chain fragments (polarons) were mobile. Further potential elevation reduced the conductivity, which reached almost zero value when the polymer was fully oxidized. This indicated that the decrease of polaron mobility was due to the lower density of unoccupied states with energy reaching a state that was suitable for hopping transports [23]. The backward scan of the voltammogram reduction of the polymer was accompanied by conductivity growth until the reduction peak potential was reached. Then, the conductivity decreased while the peak potential moved in a cathodic direction. Conductivity of the completely reduced (dedoped) polymer fell below the detection limit, which corresponded to an absence of mobile charge carriers (Figure 7). Voltammograms and conductivity-voltage profiles were reproducible from cycle to cycle, indicating the stability of the polymer film.

The electrode with the polymer film was removed from the cell after keeping it at 0.55 V, dried out and subjected to the heat treatment as described above. Then, it was placed back into the electrolyte solution. On the first CV scan of the treated film, an oxidation current showed the same magnitude as the pristine film, confirming that the polymer was dedoped (reduced) during heating and may be fully oxidized again. The charge consumed on the first oxidation cycle of the annealed polymer was even higher than the reversible reduction/oxidation charge of the pristine polymer. Thus, the thermal dedoping level was high enough to affect a “trapped” charge as well as the charge. The shape of the subsequent cycles of voltammetric curves of the heat-treated film reflected its structural changes. The width of the voltammetric peak at half of the peak current was higher for the pristine polymer than for the heat-treated film, which indicated a shortening of the delocalization length of the oxidized polymer chain [24]. The more charged states that were delocalized, the higher the conductance of the polymer [25]. The conductivity of the polymer after heating resembled the initial potential profile; however, the maximal values of conductivity decreased by four times. This can be explained by the change of morphology after annealing, which affected the charge diffusivity [23]. The decrease in conductivity was more pronounced on a negative scan of the voltammogramm. Such hysteresis was observed for polyaniline and polythiophenes and was attributed to an increase of the Coulombic repulsion between charged sites, which lowered their mobility [25]. In the case of polyNiMeOSalen, the decrease in conductivity may have resulted from the conformational change in the polymer chain after annealing, which made the polymer more ordered and decreased the interchain distance. As a result, the level of energy disorders, induced by the electric field of ionic dopants, increased at high concentrations of dopants in annealed films. This, in turn, significantly decreased the charge mobility, as suggested in [23].

## 4. Conclusions

The electrical resistivity of the conducting polymer, polyNiMeOSalen, doped with BF_4_^−^, increased with rising temperatures. This effect was studied using thermogravimetric methods and in situ conductivity measurements. While being heated to a temperature of 150 °C, it was observed that the polymer underwent the gradual loss of ca. 5% mass and increased in electrical conductivity. This temperature is an inflection point, after which a faster loss of mass and a smoother increase in resistance began. At 220 °C, the polymer resistance increased and gained two orders of magnitude when heated from 220 °C to 250 °C. This change in electrical resistance is attributed to the thermal dedoping process, which has been confirmed using EDX and XPS. However, polymer destruction did not occur, and the electrochemical activity and conductivity was restored by immersing the polymer into the electrolyte solution followed by electrochemical oxidation (doping). This feature is important for future applications of thermoresistive polymers as reversible thermosensitive circuit breakers in electrochemical systems such as Li-ion batteries.

## Supplementary Materials

The following are available online at https://www.mdpi.com/2073-4360/12/12/2925/s1, Figure S1: ATR-FTIR spectra of the polymers before and after heat treatment.
