# Supplementary material for: Resistivity-Temperature Behavior of Intrinsically Conducting Bis(3-methoxysalicylideniminato)nickel Polymer
Source: Polymers (Basel). 2020 Dec 6;12(12):2925. doi: 10.3390/polym12122925 (PMC7762270; doi:10.3390/polym12122925)
Supplement: Supplementary file 1 [file polymers-12-02925-s001.pdf]

Supplementary materials for:

Resistivity-Temperature Behavior of Intrinsically Conducting Bis(3-methoxysalicylideniminato)nickel Polymer

Evgeniy Beletskii[1], Valentin Ershov[1], Stepan Danilov[1], Daniil Lukyanov[1], Elena Alekseeva[1] and Oleg Levin[1*]

[1] St. Petersburg State University, 199034, Russian Federation, Saint Petersburg;
* Correspondence: o.levin@spbu.ru; Tel: +7-812-428-69-00

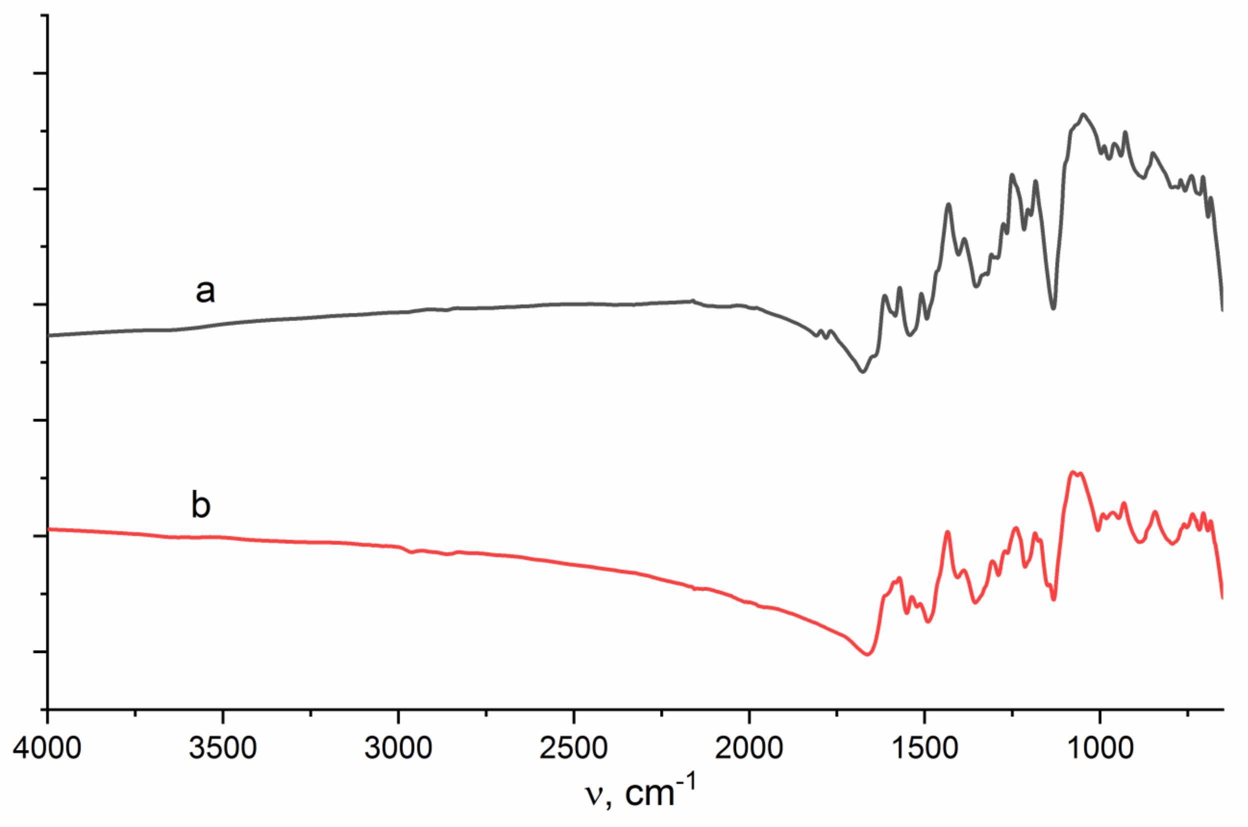

Figure S1. ATR-FTIR spectra of the initial (curve a) and heat-treated (curve b) films of polyNiMeOSalen on a GC substrate.
